# Impact of Adverse Childhood Experiences on Intimate Partner Violence Perpetration among Sri Lankan Men

**DOI:** 10.1371/journal.pone.0136321

**Published:** 2015-08-21

**Authors:** Ruvani W. Fonseka, Alexandra M. Minnis, Anu Manchikanti Gomez

**Affiliations:** 1 School of Public Health, University of California Berkeley, Berkeley, California, United States of America; 2 School of Social Welfare, University of California Berkeley, Berkeley, California, United States of America; 3 Division of Global Health, RTI International, San Francisco, California, United States of America; Örebro University, SWEDEN

## Abstract

In Sri Lanka, over one in three women experience intimate partner violence (IPV) victimization in their lifetime, making it a serious public health concern. Adverse childhood experiences (ACEs) such as child abuse and neglect, witnessing domestic violence, parental separation, and bullying are also widespread. Studies in Western settings have shown positive associations between ACEs and IPV perpetration in adulthood, but few have examined this relationship in a non-Western context. In the present study, we examined the association of ACEs with IPV perpetration among Sri Lankan men surveyed for the UN Multi-Country Study on Men and Violence in Asia and the Pacific. We found statistically significant positive associations between the number of ACE categories (ACE score) and emotional, financial, physical, and sexual IPV perpetration among Sri Lankan men. We analyzed the contributions of each ACE category and found that childhood abuse was strongly associated with perpetration of IPV in adulthood, with sexual abuse associated with the greatest increase in odds of perpetration (Adjusted odds ratio 2.36; 95% confidence interval: 1.69, 3.30). Witnessing abuse of one’s mother was associated with the greatest increase in the odds of perpetrating physical IPV (AOR 1.82; 95% CI: 1.29, 2.58), while lack of a male parental figure was not associated with physical IPV perpetration (AOR 0.76; 95% CI: 0.53, 1.09). These findings support a social learning theory of IPV perpetration, in which children who are exposed to violence learn to perpetrate IPV in adulthood. They also suggest that in Sri Lanka, being raised in a female-headed household does not increase the risk of IPV perpetration in adulthood compared to being raised in a household with a male parental figure. The relationship between being raised in a female-headed household (the number of which increased dramatically during Sri Lanka’s recent civil war) and perpetration of IPV warrants further study. Interventions that aim to decrease childhood abuse in Sri Lanka could both protect children now and reduce IPV in the future, decreasing violence on multiple fronts.

## Introduction

### Intimate Partner Violence

Intimate partner violence (IPV) victimization is the most common form of violence facing women globally [1]. In 2011 the World Health Organization (WHO) Multi-Country Study on Women’s Health and Domestic Violence found that women experienced IPV in their lifetimes at proportions ranging from 15% in Japan to 71% in Ethiopia [2]. IPV victimization is linked to a broad array of poor health outcomes among women, including anemia, being underweight, decreased sexual health, poor pregnancy outcomes, and depression [3–11]. Lowering the prevalence of IPV perpetration globally is an important public health and human rights concern.

IPV is particularly widespread in South Asia. For example, in a study of six countries across multiple world regions, Indian men had the least equitable gender attitudes, and 37 percent reported having perpetrated IPV at some point in their lifetime [12]. More than one in three married men reported having perpetrated IPV in the previous 12 months in Bangladesh [6]. In Pakistan over one third of married women reported experiencing IPV in the previous year [13]. Cultural acceptance of IPV in South Asia begins early—in Nepal and Bangladesh, more than one quarter of surveyed adolescent males condoned IPV perpetration, as did more than half of those surveyed in India [14]. In order to develop preventive interventions, it is crucial that researchers investigate the high cultural acceptance of IPV in the region, and identify the methods by which cultural acceptance, victimization, and perpetration of IPV are transmitted from one generation to the next.

A 2014 World Bank report on global violence against women and girls stressed the need for more research to establish the effect of risk and protective factors related to IPV in South Asia, specifically highlighting the lack of Sri Lanka-focused research [15]. Multiple studies estimated the prevalence of IPV victimization in Sri Lanka to range from 34 to 40 percent, nearly identical to those of its South Asian neighbors, but these studies were geographically limited to single regions or cities [16–18]. In one study examining cultural acceptance of IPV, more than half of both male and female Sri Lankan medical students justified wife beating and agreed that women were responsible for any IPV they experienced [19]. Sri Lankan laws reinforce this cultural acceptance of IPV by permitting sexual IPV perpetration, including rape, within marriage [18].

### Adverse Childhood Experiences and IPV

Many researchers have identified a relationship between adverse childhood experiences (ACEs) and IPV victimization, primarily linking violent family experiences, such as witnessing abuse of one’s mother, to negative psychosocial outcomes in children and IPV victimization among adult women [20–26]. In addition, the US ACE study revealed a significant and positive relationship between ACEs and perpetration of IPV in adulthood [27]. In the original US study, ACE categories included childhood emotional, physical, and sexual abuse and other items capturing household dysfunction, including parental separation or divorce, having a caregiver in prison, witnessing abuse of one’s mother, and living with a mentally ill family member [28]. Over time, this list was expanded to include physical and emotional neglect in childhood [27]. Other researchers have added non-household factors such as peer victimization or bullying [29]. In addition to IPV, exposure to a large number of ACE categories has been linked to poor health outcomes in adulthood, as well as health risk behaviors, such as drug use and alcoholism [27,28,30]. US researchers have also linked perpetration of IPV to individual ACE categories, such as childhood physical abuse and witnessing abuse of one’s mother [23,31–35]. In South Africa and India, and in some multi-national studies, researchers have identified positive relationships between ACEs and IPV perpetration among men, particularly childhood abuses and witnessing abuse of one’s mother in childhood [12,20,24,34,35]. Many researchers have explained these relationships using Bandura’s social learning theory, which posits that people learn behaviors they are exposed to and perceive to be rewarded by their environment [36]. However, as a review by Schumacher, et al. points out, studies examining IPV perpetration risk factors frequently do not consider whether the ACEs experienced were unique or overlapping [37]. This approach overlooks the fact that ACEs often co-occur and might not be separable as risk factors for IPV. The numerous findings linking ACEs to perpetration of IPV are promising for IPV prevention efforts across the world, as they indicate that reducing and addressing the impact of ACEs could also reduce perpetration of IPV.

There is currently very little research on the relationship between ACEs and IPV perpetration in Sri Lanka. Sri Lanka recently ended a 30-year civil war, which exposed the population to widespread armed conflict and left an unprecedented number of female-headed households in the country. In the North and East provinces alone, the war resulted in over 90,000 widows, whose children were exposed to the ACE of lacking a male parental figure [38]. Jayasinghe, et al. found that witnessing abuse of one’s mother was associated with mental illness in children, a potential precursor of IPV perpetration and victimization in adulthood [39]. Haj-Yahia and de Zoysa showed that Sri Lankan medical students who were exposed to family violence in childhood were more likely to blame and less likely to help women experiencing IPV [19].

The first study to focus on perpetration of IPV in Sri Lanka was the UN Multi-Country Study on Men and Violence in Asia and the Pacific from 2011–2012 [40–42]. The researchers found positive associations between some individual ACE categories, including childhood abuse and witnessing abuse of one’s mother; and physical and sexual IPV perpetration [28,40,42]. The present study builds on these results, and, like the US ACE study, evaluates the association between cumulative ACEs and IPV perpetration [37]. Given that ACEs are often co-occurring, research on the impact of multiple ACEs on IPV perpetration in Sri Lanka can help us understand how to prevent IPV and to better identify young people most at risk for IPV perpetration later in life.

### Present Research Aims and Hypotheses

Our study aimed to explore the relationship between Sri Lankan men’s ACEs and perpetration of emotional, financial, physical, and sexual IPV in adulthood. To accomplish this, we tested the following two hypotheses:

1. Sri Lankan men’s ACE scores (the number of cumulative ACE categories an individual has experienced) have a positive dose-response relationship with their likelihood of perpetrating IPV, similar to the relationship found in the US ACE study [27].
2. Individual ACE categories, particularly childhood abuse and witnessing abuse of one’s mother, are positively associated with IPV perpetration among Sri Lankan men.

This analysis aimed to contribute to a greater understanding of the relationship between ACEs and perpetration of IPV in Sri Lanka, South Asia, and other post-conflict areas around the world. In finding that high ACE scores are linked to IPV perpetration in Sri Lanka, our research in part helps explain the high levels of IPV practices and societal acceptance of IPV in Sri Lanka through intergenerational transmission and childhood trauma. A better understanding of the contribution of ACEs to adulthood IPV perpetration can lead to the successful prevention of IPV in the future by derailing the generational cycle of violence.

## Methods

### Study Design and Sample

**Fig 1:**
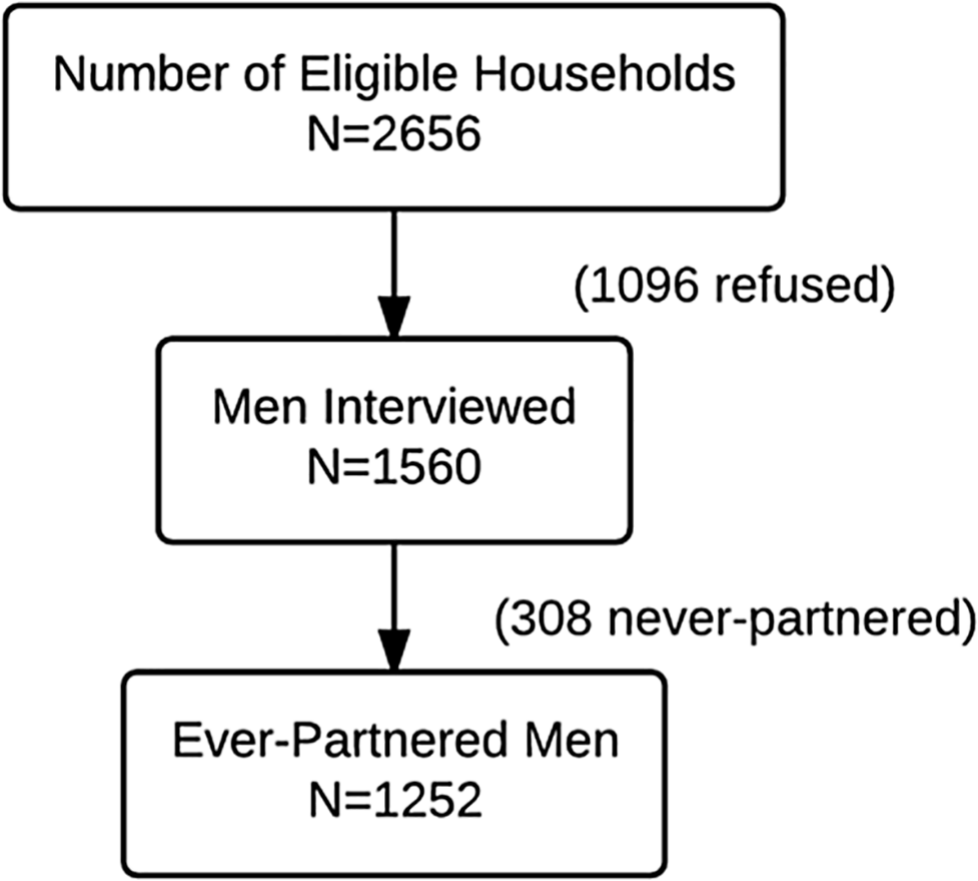
Sri Lankan Men’s eligibility and participation by ever being partnered, UN Multi-Country Study on Men and Violence in Asia and the Pacific, Sri Lanka, 2011–2012

CARE International Sri Lanka, a non-governmental organization with expertise working on gender-based violence, collected the data used in this analysis for the UN Multi-Country Study on Men and Violence in Asia and the Pacific [43]. CARE and Partners for Prevention (a UN joint program for the prevention of violence against women and girls in Asia and the Pacific) trained male interviewers fluent in Sri Lanka’s two most common languages, Sinhala or Tamil, over a seven-day session focused on four areas: 1) gender and masculinity, 2) the questionnaire, 3) handing the personal digital assistant (PDA) devices used to administer the survey, and 4) field techniques and research ethics [42]. Using a multistage cluster sampling procedure, interviewers approached 2656 eligible households and surveyed 1560 men (1096 households did not agree to participate) between the ages of 18 and 49 years (see Fig 1) throughout 4 of the 25 districts of Sri Lanka. The four districts surveyed were: Colombo, the country’s capital and largest city; Hambantota, a southern district that experienced the 2004 tsunami; Batticaloa, an eastern district affected both by the tsunami and recent civil war; and Nuwara Eliya, a central district where tea plantations are the major industry [40]. Data collection took place between January 2011 and December 2012. For the present analysis, our analytical sample included the 1252 men who reported a current and/or past intimate relationship and were therefore considered “ever-partnered.”

A multi-national team of gender research experts developed a standardized structured questionnaire derived from internationally recognized tools, including the International Men and Gender Equality Survey (IMAGES), the questionnaire used in the WHO Multi-Country Study on Women’s Health and Domestic Violence, and the South African Medical Research Council’s Men’s Health and Relationships Study [9,12]. The questionnaire was tested for reliability and validity using cognitive interviews. The validity was supported by high Cronbach’s α values for IPV measures and other scales [40]. The trained interviewers administered the surveys in the participants’ homes, using PDAs to record the answers. To ensure maximum confidentiality and reduce biases associated with self-reporting sensitive information, respondents recorded answers themselves on the PDAs for the most sensitive questions—those related to sexual practices and sexual violence, current mental health, and socio-economic status indicators—without the interviewer being privy to their responses. For participants who could not read, an audio feature on the PDA enabled self-administration. The data collection methods are described in detail in the study report by CARE International and Partners for Prevention and in articles summarizing the UN Multi-Country Study on Men and Violence in Asia and the Pacific [40–44].

### Ethics Statement

The UN Multi-Country Study followed the World Health Organization’s ethical guidelines for research, and Sri Lankan and international researchers obtained ethical clearance for both the research instrument and the methods from the Sri Lanka Medical Association (SLMA) Ethical Review Committee. Due to the sensitive nature of the questions, verbal consent was obtained from the participants to ensure participant anonymity. De-identified data were used in this secondary data analysis, which the University of California, Berkeley Committee for the Protection of Human Subjects deemed exempt from review. CARE International Sri Lanka is the sole owner of the data set, which was made available to the authors. Other researchers interested in using the same data should contact CARE International Sri Lanka at srilanka@co.care.org.

### Key Measures

#### Dependent Variables/Outcomes of Interest: IPV Perpetration

We focused our research on the lifetime perpetration of different forms of IPV by Sri Lankan men. Following the example of Fulu, et al., we focused on adulthood lifetime prevalence to add more power to the analysis [40]. The participants replied to questions about the frequency of specific acts of IPV perpetration using a 4-point Likert-type scale ranging from “never” to “many” times. The survey asked about perpetration of the following four forms of IPV:

1. Emotional IPV: five questions, including “have you ever threatened to hurt a partner?”
2. Financial IPV: four questions, including “have you ever prohibited a partner from earning money?”
3. Physical IPV: five questions, including “have you ever pushed or shoved a partner?”
4. Sexual IPV: two questions, including “have you ever forced your current or previous partner to have sex with you when she did not want to?”

We scored responses of “never” as 0 and all other responses as 1. If a respondent had committed one or more acts within a form of IPV, we counted them as ever having perpetrated that form of IPV in our analysis. Additionally, we created a binary summary variable, “Any IPV,” which captured whether a respondent had perpetrated any of the four forms of IPV.

#### Independent Variables/Exposures of Interest: ACEs

We investigated the association of perpetration of IPV with Sri Lankan men’s ACE categories, which were captured using a modified version of the Childhood Trauma Questionnaire of which Bernstein, et al. demonstrated the reliability and validity [45,46]. In addition to seven dichotomous ACE categories that were analogous to ACE categories used in the US ACE study, we included a measure of peer victimization based on research by Finkelhor, et al. showing the importance of ACEs outside of the home in the US [27,29]. The UN Multi-Country Study on Men and Violence in Asia and Pacific, while extensive, did not include questions about two ACE categories in the US ACE study: living with a family member with mental illness during childhood; and growing up with one or more incarcerated family member. We scored all ACE categories, with the exception of lack of a male parental figure, from statements about the men’s childhood that they replied to using a four-point Likert-type scale, ranging from “never” to “very often.” Replicating the methods of the US ACE study, we scored item responses of “never” as 0 and all other responses as 1 [28]. If a respondent reported one or more experiences within an ACE category, we counted them as having that ACE category in our analysis. We used the following eight ACE categories:

1. Childhood emotional abuse: two items, including “Before I reached 18, I was told I was lazy or stupid or weak by someone in my family.”
2. Childhood physical abuse: three items, including “Before I reached 18, I was beaten so hard at home that it left a mark or bruise.”
3. Childhood sexual abuse: six items, including “Before I reached 18, I was exposed to unwanted incidents of a sexual nature.”
4. Childhood hunger: one item, “Before I reached 18, I did not have enough to eat.”
5. Childhood neglect::::;l: three items, including “Before I reached 18, one or both of my parents were too drunk or drugged to take care of me.”
6. Witnessing abuse of one’s mother: one item, “Before I reached 18, I saw or heard my mother being beaten by her husband or boyfriend.”
7. Lack of a male parental figure: scored using an answer of “never at home” or “rarely at home” in response to the question, “When you were growing up, would you say that your biological father was…”; and a “no” response to the question, “Apart from your biological father, were there other important male figures in your life when you were growing up?” The UN Multi-Country Study on Men and Violence in Asia and the Pacific did not ask participants directly about parental divorce or separation, which is uncommon and stigmatized in Sri Lanka, so this was our closest approximation of the “parental separation or divorce” category from the US ACE study [27,42].
8. Peer victimization: one item, “Were you bullied, teased, or harassed in school or in the neighborhood in which you grew up?”

Finally, we created an “ACE Score” variable using an identical method to that of the US ACE study researchers–we added the dichotomous scores of each ACE category to record an overall ACE Score for each individual that ranged from 0 to 8.

#### Other Variables of Interest (Possible Confounders)

**Fig 2:**
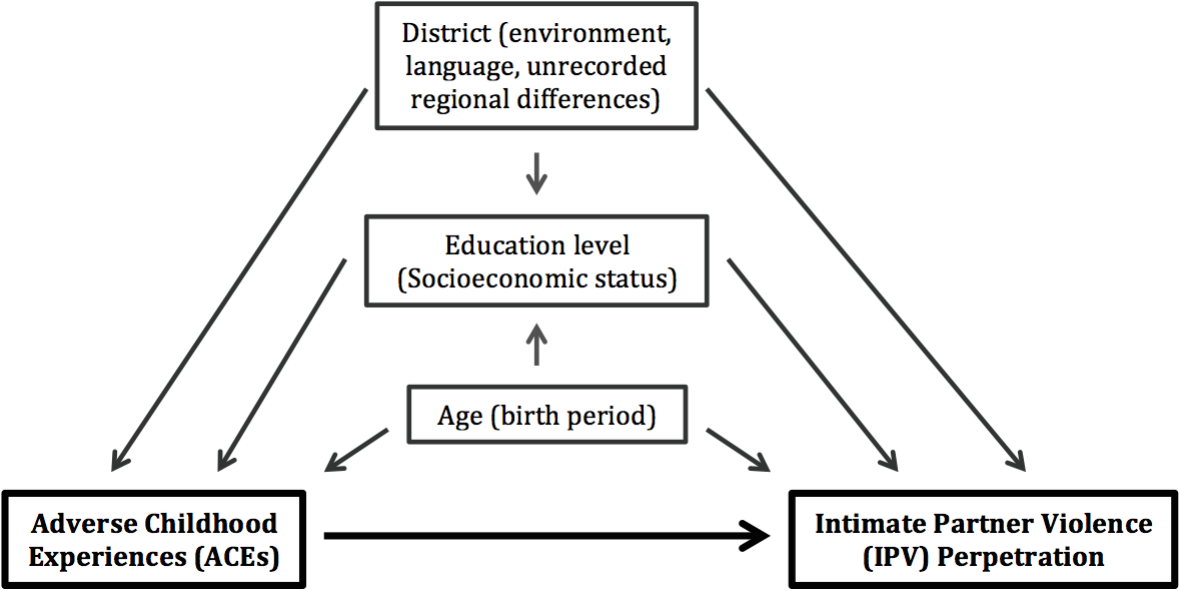
Directed acyclic graph (DAG) of the hypothesized relationship between ACEs and IPV with potential confounders, UN Multi-Country Study on Men and Violence in Asia and the Pacific, Sri Lanka, 2011–2012

We considered several demographic variables as possible confounding factors of a causal relationship between men’s ACEs and perpetration of IPV in Sri Lanka, focusing on factors that could have affected both men’s experiences of ACEs and their perpetration of IPV (Fig 2) [47]. In multivariate logistic regression models, we controlled for geographic district (Colombo, Hambantota, Batticaloa, or Nuwara Eliya) to account for rural vs. urban environments; language spoken (in each district, more than 95% of the sample spoke one language, making them near-homogeneous); and possible unknown or unrecorded differences between men’s lives in the various districts, such as regional norms. We controlled for age group (18–24, 25–34, and 35–49 years) because we expected age to affect both the outcome (lifetime prevalence of IPV perpetration, which would increase with age) and exposure, with ACEs potentially differing across age groups due to generational differences. Finally, we controlled for education (none, primary, some secondary, completed secondary, or any higher) as a marker of socioeconomic status (SES), as prior studies have suggested that SES is linked to IPV perpetration and ACE history [20,40,48,49]. Other researchers have considered items during adulthood such as alcohol abuse, empathy, and gender attitudes as potential risk factors for IPV. However, these factors could not have caused ACEs due to their timing; therefore, we did not include them in our analysis of the relationship between ACEs and IPV perpetration. If associated with ACEs and IPV, it is more likely that those factors are intermediary consequences of ACEs that are associated with IPV perpetration, rather than confounders influencing both exposure and outcome.

### Statistical analysis

We analyzed the data using Stata version 12.1. In our descriptive analysis, we examined the basic features of the sample, including their distribution by district, age, education, and language. We determined the prevalence of each ACE category and form of IPV perpetration. To allow for comparison with the US ACE study, we analyzed the relationships between different ACE categories and calculated the prevalence of ACE scores greater than or equal to 4 for each ACE category, replicating a cut-point used in the US ACE Study [28]. Finally, we examined the distribution of each form of IPV perpetration among men exposed to each ACE category.

We then developed multivariate logistic regression models to study the relationship between ACEs and the five IPV outcome variables (each of the four individual IPV forms and the overall IPV perpetration measure). We adjusted for district, age group, and education in all models. Our first set of models used the ACE score as the independent variable to look for evidence of positive dose-response relationships between ACEs and IPV perpetration. Our final set of five regression models included all eight of the ACE categories as independent variables to identify the individual contribution of each category to perpetration of our five IPV outcome variables.

## Results

### Descriptive Results

#### Demographic Factors

We first examined the demographic characteristics of our sample of ever-partnered men (Table 1). The four surveyed districts each contributed between 20 and 35 percent of our sample. Most of the sample left school before finishing their secondary education (63%), and over half spoke Tamil (53%), although Sinhala is spoken by a majority of Sri Lanka’s population [50]. This imbalance was a result of the districts surveyed–Colombo and Hambantota are majority Sinhalese, while Batticaloa and Nuwara Eliya are majority Tamil. Overall, the data came from a broad sample of different populations in Sri Lanka. While not representative of the country as a whole, the sample was deemed by the survey architects to be representative of the four surveyed districts, which covered a variety of regions in the country [40].

**Table 1:** Demographic characteristics of sample of ever-partnered Sri Lankan men (n = 1252), UN Multi-Country Study on Men and Violence in Asia and the Pacific, Sri Lanka, 2011–2012

Characteristic	Prevalence	
	n	%
**Overall**	1252	100
**District**		
Colombo	258	20.6
Hambantota	321	25.6
Batticaloa	269	21.5
Nuwara Eliya	404	32.3
**Age (years)**		
18–24	271	21.7
25–34	460	36.7
35–49	521	41.6
**Educational attainment**		
None	16	1.3
Primary	121	9.7
Some secondary	651	52.0
Completed secondary	339	27.1
Any higher	124	9.9
**Language**		
Sinhala	585	46.7
Tamil	667	53.3

#### Prevalence of Independent and Dependent Variables

Childhood physical abuse was the most prevalent ACE category, with more than half (59%) of men reporting being beaten before the age of 18, and nearly half of all men (48%) reporting having experienced physical abuse by an authority figure at school (Table 2). The category of peer victimization was experienced least: only 17 percent of men reported being bullied, teased or harassed in their school or neighborhood while growing up. ACEs were widespread and co-occurring, with almost one third of men reporting having experienced 4 or more ACEs in their childhood (31%).

**Table 2:** Adverse childhood experience (ACE) categories and item prevalence among ever-partnered Sri Lankan men (n = 1252), UN Multi-Country Study on Men and Violence in Asia and the Pacific, Sri Lanka, 2011–2012.

Adverse childhood experience category	Item			Category		
	n	%	(95% CI) ^b^	n	%	(95% CI) ^b^
**Childhood emotional abuse** ^a^	—	—	—	**359**	**28.7**	**(26.2–31.2)**
I was told I was lazy or stupid or weak by someone in my family	286	22.8	(20.5–25.1)	—	—	—
I was insulted or humiliated by someone in my family in front of other people	222	17.7	(15.6–19.8)	—	—	—
**Childhood physical abuse** ^a^	—	—	—	**742**	**59.3**	**(56.6–62.0)**
I was beaten at home with a belt or stick or whip or something else which was hard	458	36.5	(33.8–39.2)	—	—	—
I was beaten so hard at home that it left a mark or bruise	166	13.3	(11.4–15.2)	—	—	—
I was beaten or physically punished at school by a teacher or headmaster	604	48.2	(45.4–51.0)	—	—	—
**Childhood sexual abuse** ^a^	—	—	—	**340**	**27.2**	**(24.7–29.7)**
Someone touched my buttocks or genitals or made me touch them when I did not want to	156	12.5	(10.7–14.3)	—	—	—
I had sex with a woman who was more than five years older to me	77	6.2	(4.9–7.5)	—	—	—
I had sex with someone because I was threatened or frightened or forced	46	3.7	(2.7–4.8)	—	—	—
I was exposed to unwanted incidents of a sexual nature	133	10.6	(8.9–12.3)	—	—	—
I was forced to have sex or physical relations with a community leader/ older schoolboy	40	3.2	(2.2–4.2)	—	—	—
I was exposed to pornographic material against my will	187	14.9	(12.9–16.9)	—	—	—
**Childhood hunger** ^a^	—	—	—	**476**	**38.0**	**(35.3–40.7)**
I did not have enough to eat	476	38.0	(35.3–40.7)	—	—	—
**Childhood neglect** ^a^	—	—	—	**475**	**37.9**	**(35.2–40.6)**
I lived in different households at different times	327	26.1	(23.7–28.5)	—	—	—
One or both of my parents were too drunk or drugged to take care of me	132	10.6	(8.9–12.3)	—	—	—
I spent time outside the home and none of the adults at home knew where I was	207	16.5	(14.4–18.6)	—	—	—
**Witnessed abuse of mother** ^a^	—	—	—	**367**	**29.3**	**(26.8–31.8)**
I saw or heard my mother being beaten by her husband or boyfriend	367	29.3	(26.8–31.8)	—	—	-
**Lack of a male parental figure**	—	—	—	**324**	**25.9**	**(23.5–28.3)**
When you were growing up, would you say that your biological father was present?	437	34.9	(32.3–37.5)	—	—	—
Apart from your biological father, were there other important male figures in your life when you were growing up?	943	75.3	(72.9–77.7)	—	—	—
**Peer victimization**	—	—	—	219	**17.5**	**(15.4–19.6)**
Were you bullied, teased or harassed in school or in the neighborhood in which you grew up?	219	17.5	(15.4–19.6)	—	—	—
**Four or more ACE categories**	—	—	—	393	**31.4**	**(28.8–34.0)**

^a^All questions in this category were preceded by the phrase “Before I reached 18…”;

^b^95% confidence interval

In addition to commonly reporting ACEs, almost half of all the men (49%) reported having perpetrated at least one form of IPV at some point in their lifetime (Table 3). Emotional IPV perpetration (37%) was most common, with more than one quarter (28%) of men reporting having purposefully scared or intimidated a partner. Sexual IPV was the least commonly reported form of IPV perpetration (14%). The least-reported individual IPV perpetration items (both 2%) were forms of physical IPV: kicking, dragging, beating, choking or burning a partner; and threatening to use or using a gun against a partner.

**Table 3:** Intimate partner violence (IPV) perpetration forms and item prevalence among ever-partnered Sri Lankan men (n = 1252), UN Multi-Country Study on Men and Violence in Asia and the Pacific, Sri Lanka, 2011–2012.

Form of intimate partner violence perpetration	Item			IPV form		
	n	%	(95% CI) ^a^	n	%	(95% CI)^a^
**Emotional intimate partner violence perpetration**	**—**	**—**	—	**460**	**36.7**	**(34.0–39.4)**
Have you ever insulted a partner or deliberately made her feel bad about herself?	167	13.3	(11.4–15.2)	—	—	—
Have you ever belittled or humiliated a partner in front of other people?	122	9.7	(8.1–11.3)	—	—	—
Have you ever done things to scare or intimidate a partner on purpose for example by the way you looked at her, by yelling and smashing things?	353	28.2	(25.7–30.7)	—	—	—
Have you ever threatened to hurt a partner?	204	16.3	(14.3–18.4)	—	—	—
Have you ever hurt people your partner cares about as a way of hurting her, or damaged things of importance to her?	110	8.8	(7.2–10.4)	—	—	—
**Financial intimate partner violence perpetration**	—	—	—	**204**	**16.3**	**(14.3–18.4)**
Have you ever prohibited a partner from getting a job, going to work, trading or earning money?	103	8.2	(6.7–9.7)	—	—	—
Have you ever taken a partner’s earnings against her will?	67	5.4	(4.2–6.7)	—	—	—
Have you ever thrown a partner out of the house?	58	4.6	(3.4–5.8)	—	—	—
Have you ever kept money from your earnings for alcohol, tobacco or other things for yourself when you knew your partner was finding it hard to afford the household expenses?	92	7.4	(6.0–8.9)	—	—	—
**Physical intimate partner violence perpetration**	—	—	—	**274**	**21.9**	**(19.6–24.2)**
Have you ever slapped a partner or thrown something at her that could hurt her?	144	11.5	(9.7–13.3)	—	—	—
Have you ever pushed or shoved a partner?	216	17.3	(15.2–19.4)	—	—	—
Have you ever hit a partner with a fist or with something else that could hurt her?	77	6.2	(4.9–7.5)	—	—	—
Have you ever kicked, dragged, beaten, choked or burned a partner?	28	2.2	(1.4–3.0)	—	—	—
Have you ever threatened to use or actually used a gun, against a partner?	25	2.0	(1.2–2.8)	—	—	—
**Sexual intimate partner violence perpetration**	—	—	—	**171**	**13.7**	**(11.8–15.6)**
Have you ever forced your current or previous wife or girlfriend to have sex with you when she did not want to?	58	4.6	(3.4–5.8)	—	—	—
Have you ever had sex with your current or previous wife or girlfriend when you knew she didn’t want it but you believed she should agree because she was your wife/partner?	115	9.2	(7.6–10.8)	—	—	—
**Perpetration of any form of intimate partner violence**	—	—	—	**617**	**49.3**	**(46.5–52.1)**

^a^95% confidence interval

#### Relationships between ACE Categories

To test the relevance of the ACE framework in Sri Lanka, we examined relationships between ACE categories (Table 4), replicating the methods used in the US ACE study [28]. Almost all of the bivariate distributions were statistically significant (p≤.05; chi square). However, lack of a male parental figure was significantly associated only with the distribution of emotional abuse.

**Table 4:** Relationships between adverse childhood experience (ACE) categories among ever-partnered Sri Lankan men (n = 1252), UN Multi-Country Study on Men and Violence in Asia and the Pacific, Sri Lanka, 2011–2012.

	Amount exposed to another ACE category																										
First ACE category	Childhood emotional abuse			Childhood physical abuse			Childhood sexual abuse			Childhood hunger			Childhood neglect			Witnessed abuse of mother			Lack of a male parental figure			Peer victimization			ACE score ≥ 4		
	n	%	(95% CI)^a^	n	%	(95% CI)^a^	n	%	(95% CI)^a^	n	%	(95% CI)^a^	n	%	(95% CI)^a^	n	%	(95% CI)^a^	n	%	(95% CI)^a^	n	%	(95% CI)^a^	n	%	(95% CI) ^a^
**Sample prevalence**	**359**	**28.7**	**(26.2–31.2)**	**742**	**59.3**	**(56.6–62.0)**	**340**	**27.2**	**(24.7–29.7)**	**476**	**38.0**	**(35.3–40.7)**	**475**	**37.9**	**(35.2–40.6)**	**367**	**29.3**	**(26.8–31.8)**	**324**	**25.9**	**(23.5–28.3)**	**219**	**17.5**	**(15.4–19.6)**	**393**	**31.4**	**(28.8–34.0)**
**Childhood emotional abuse**	—	—	—	285	79.4**	(77.1–81.6)	174	48.5**	(45.7–51.3)	204	56.8**	(54.1–59.5)	225	62.7**	(60.0–65.4)	190	52.9**	(50.1–55.7)	115	32.0*	(29.4–34.6)	128	35.7**	(33.1–38.4)	253	70.5**	(68.0–73.0)
**Childhood physical abuse**	285	38.4**	(35.7–41.1)	—	—	—	285	38.4**	(35.7–41.1)	345	46.5**	(43.7–49.3)	368	49.6**	(46.8–52.4)	305	41.1**	(38.4–43.8)	177	23.9	(21.5–26.3)	170	22.9**	(20.6–25.2)	364	49.1**	(46.3–51.9)
**Childhood sexual abuse**	174	51.2**	(48.4–54.0)	285	83.8**	(81.8–85.8)	—	—	—	180	52.9**	(50.1–55.7)	228	67.1**	(64.5–69.7)	172	50.6**	(47.8–53.4)	106	31.2	(28.6–33.8)	103	30.3**	(27.8–32.9)	254	74.7**	(72.3–77.1)
**Childhood hunger**	204	42.9**	(40.2–45.6)	345	72.5**	(70.0–75.0)	180	37.8**	(35.1–40.5)	—	—	—	265	55.7**	(53.0–58.5)	221	46.4**	(43.6–49.2)	128	26.9	(24.4–29.4)	131	27.5**	(25.0–30.0)	273	57.4**	(54.7–60.1)
**Childhood neglect**	225	47.4**	(44.6–50.2)	368	77.5**	(75.2–79.8)	228	48.0**	(45.2–50.8)	265	55.8**	(53.1–58.6)	—	—	—	238	50.1**	(47.3–52.9)	130	27.4	(24.9–29.9)	126	26.5**	(24.1–28.9)	309	65.1**	(62.5–67.7)
**Witnessed abuse of mother**	190	51.8**	(49.0–54.6)	305	83.1**	(81.0–85.2)	172	46.9**	(44.1–49.7)	221	60.2**	(57.5–62.9)	238	64.9**	(62.3–67.5)	—	—	—	93	25.3	(22.9–27.7)	103	28.1**	(25.6–30.6)	263	71.7**	(69.2–74.2)
**Lack of a male parental figure**	115	35.5*	(32.9–38.2)	177	54.6	(51.8–57.4)	106	32.7	(30.1–35.3)	128	39.5	(36.8–42.2)	130	40.1	(37.4–42.8)	93	28.7	(26.2–31.2)	—	—	—	72	22.2	(19.9–24.5)	142	43.8**	(41.1–46.6)
**Peer victimization**	128	58.5**	(55.8–61.2)	170	77.6**	(75.3–79.9)	103	47.0**	(44.2–49.8)	131	59.8**	(57.1–62.5)	126	57.5**	(54.8–60.2)	103	47.0**	(44.2–49.8)	72	32.9**	(30.3–35.5)	—	—	—	152	69.4**	(66.9–72.0)
**ACE score** ≥ **4**	253	64.4**	(61.8–67.1)	364	92.6**	(91.2–94.1)	254	64.6**	(62.0–67.3)	273	69.5**	(67.0–72.1)	309	78.6**	(76.3–80.9)	263	66.9**	(64.3–69.5)	142	36.1**	(33.4–28.8)	152	38.7**	(36.0–41.4)	—	—	—

*Identifies a chi-squared test statistic with p≤.05

**Identifies a chi-squared test statistic with p≤.001

^a^95% confidence interval

Nearly a third of respondents (31%) reported four or more ACE categories. Among these respondents, almost all (93%) had experienced childhood physical abuse. ACE categories often overlapped, and men reporting other ACE categories frequently reported childhood physical abuse. Men who reported childhood sexual abuse and men who witnessed abuse of their mother reported childhood physical abuse as an additional ACE most frequently (84 and 83% respectively). Lack of a male parental figure and peer victimization were the ACE categories least frequently reported as accompanying other ACE categories, but were still reported by one in five respondents reporting another ACE category (22–39%). These associations between various ACE categories highlight the importance of analyzing the impact of ACE categories in combination, and the cumulative burden of ACEs on individual men in Sri Lanka.

#### Relationships between IPV Perpetration, ACEs and Covariates

We calculated the prevalence of IPV perpetration among men who had experienced each ACE category and calculated the significance of the association (p≤.05; chi-square analysis) between each ACE category and each IPV outcome variable (Table 5). The proportions of almost all forms of IPV perpetration were significantly higher among men experiencing each ACE category compared to those who had not, except for lack of a male parental figure, which only had a statistically significant association with perpetration of emotional IPV. Childhood sexual abuse was significantly associated with the greatest proportions of perpetration of emotional (58%), sexual (26%) and any IPV (75%). Childhood emotional abuse was significantly associated with the highest proportion of perpetration of financial IPV (31%), and peer victimization was significantly associated with the highest proportion of perpetration of physical IPV (40%).

**Table 5:** Prevalence of intimate partner violence (IPV) perpetration across adverse childhood experience (ACE) categories and selected covariates among ever-partnered Sri Lankan men (n = 1252), UN Multi-Country Study on Men and Violence in Asia and the Pacific, Sri Lanka, 2011–2012.

			Percent (%) perpetrating IPV														
ACE category/covariate	Sample Prevalence		Emotional IPV			Financial IPV			Physical IPV			Sexual IPV			Any form of IPV		
	n	%	n	%	(95% CI)	n	%	(95% CI)	n	%	(95% CI)	n	%	(95% CI)	n	%	(95% CI)
**Sample prevalence**	**—**	**—**	**460**	**36.7**	**(34.0–39.4)**	**204**	**16.3**	**(14.3–18.4)**	**274**	**21.9**	**(19.6–24.2)**	**171**	**13.7**	**(11.8–15.6)**	**617**	**49.3**	**(46.5–52.1)**
**Childhood emotional abuse**	359	28.7	196	57.8**	(55.1–60.5)	104	30.7**	(28.2–33.3)	131	38.2**	(35.5–40.9)	74	20.6**	(18.4–22.8)	245	71.2**	(68.7–73.7)
**Childhood physical abuse**	742	59.3	340	47.9**	(45.1–50.7)	141	19.9*	(17.7–22.1)	211	29.8**	(27.3–32.3)	140	18.9**	(16.7–21.1)	450	63.2**	(60.5–65.9)
**Childhood sexual abuse**	340	27.2	194	58.3**	(55.6–61.0)	96	28.9**	(26.4–31.4)	112	33.6**	(31.0–36.2)	88	25.9**	(23.5–28.3)	252	75.2**	(72.8–77.6)
**Childhood hunger**	476	38.0	235	51.2**	(48.4–54.0)	117	25.6**	(23.2–28.0)	164	35.8**	(33.1–38.5)	90	18.9*	(16.7–21.1)	313	67.9**	(65.3–70.5)
**Childhood neglect**	475	37.9	243	52.7**	(49.9–55.5)	124	27.1**	(24.6–29.6)	139	30.2**	(27.7–32.7)	102	21.5**	(19.2–23.8)	317	68.5**	(65.9–71.1)
**Witnessed abuse of mother**	367	29.3	184	52.9**	(50.1–55.7)	88	25.7**	(23.3–28.1)	131	38.2**	(35.5–40.9)	73	19.9*	(17.7–22.1)	239	68.9**	(66.3–71.5)
**Lack of a male parental figure**	324	25.9	133	45.1**	(42.3–47.9)	58	19.5	(17.3–21.7)	71	23.8	(21.4–26.2)	42	13.0	(11.1–14.9)	165	55.2	(52.5–58.0)
**Peer victimization**	219	17.5	110	52.1**	(49.3–54.9)	59	27.8**	(25.3–30.3)	85	40.1**	(37.4–42.8)	40	18.3	(16.2–20.4)	141	66.2**	(63.6–68.8)
**ACE Score** ≥ **4**	393	31.4	219	57.6**	(54.9–60.3)	108	28.5**	(26.0–31.0)	141	37.3**	(34.6–40.0)	88	22.4**	(20.1–24.7)	276	72.6**	(70.1–75.1)
**District**																	
**Colombo**	258	20.6	11	45.9	(43.1–48.7)	35	14.6**	(12.6–16.6)	64	26.7**	(24.3–29.2)	59	22.9**	(20.6–25.2)	157	65.2*	(62.6–67.8)
**Hambantota**	321	25.6	121	39.2	(36.5–41.9)	31	10.0**	(8.3–11.7)	54	17.4**	(15.3–19.5)	57	17.8**	(15.7–19.9)	152	48.9*	(46.1–51.7)
**Batticaloa**	269	21.5	88	38.1	(35.4–40.8)	41	17.9**	(15.8–20.0)	76	33.0**	(30.4–35.6)	14	5.2**	(4.0–6.4)	126	54.1*	(51.3–56.9)
**Nuwara Eliya**	404	32.3	140	40.1	(37.4–42.8)	97	27.6**	(25.1–30.1)	80	22.8**	(20.5–25.1)	41	10.2**	(8.5–11.9)	182	51.6*	(48.8–54.4)
**Age (years)**																	
**18–24**	271	21.7	64	29.0**	(26.5–31.5)	32	14.6	(12.6–16.6)	26	11.8**	(10.0–13.6)	19	7.0**	(5.6–8.4)	94	42.5*	(39.8–45.2)
**25–34**	460	36.7	175	41.8**	(39.1–44.5)	87	20.6	(18.4–22.8)	107	25.4**	(23.0–27.8)	71	15.4**	(13.4–17.4)	241	56.8*	(54.1–59.5)
**35–49**	521	41.6	221	45.0**	(42.2–47.8)	85	17.4	(15.3–19.5)	141	28.8**	(26.3–31.3)	81	15.6**	(13.6–17.6)	282	57.2*	(54.5–59.9)
**Education**																	
**none**	16	1.3	4	28.6	(26.1–31.1)	4	28.6	(26.1–31.1)	2	14.3	(12.4–16.2)	0	0.0	N/A	5	35.7	(33.1–38.4)
**primary**	121	9.7	46	41.1	(38.4–43.8)	23	20.5	(18.3–22.7)	28	25.2	(22.8–27.6)	11	9.1	(7.5–10.7)	60	53.6	(50.8–56.4)
**some secondary**	651	52.0	234	39.3	(36.6–42.0)	115	19.3	(17.1–21.5)	148	24.9	(22.5–27.3)	97	14.9	(12.9–16.9)	319	53.4	(50.6–56.2)
**complete secondary**	339	27.1	123	41.0	(38.3–43.7)	40	13.3	(11.4–15.2)	69	22.9	(20.6–25.2)	44	13.0	(11.1–14.9)	166	54.4	(51.6–57.2)
**any higher**	124	9.9	53	49.1	(46.3–51.9)	21	19.6	(17.4–21.8)	27	24.8	(22.4–27.2)	19	15.3	(13.3–17.3)	66	60.6	(57.9–63.3)

*Identifies a chi-squared test statistic with p≤.05

**Identifies a chi-squared test statistic with p≤.001

^a^95% confidence interval

Among the selected covariates, district and age were associated with most forms of IPV perpetration, while education, which we used as a marker of SES, was not associated with any form of IPV perpetration. An increase in age was associated with an increased likelihood of perpetration of all the forms of IPV perpetration we studied except financial. Rates differed across the districts, with the most frequently reported form of IPV perpetration being emotional IPV in all four districts (38–46%). Men in Colombo most frequently reported having perpetrated any form of IPV in their lifetime (65%).

### Multivariate Results

#### Relationships between Number of ACE Categories and Perpetration of IPV

We conducted logistic regression analyses to understand the relationship between ACEs and IPV perpetration, controlling for district, age, and educational attainment. We tested for a dose-response relationship between the number of ACE categories experienced and IPV perpetration by using the men’s ACE score (0, 1, 2, 3, and 4 or more ACE categories) as the exposure in logistic regression models for each form of IPV (Table 6). Higher ACE scores were consistently linked to statistically significant increases in the odds of perpetrating all forms of IPV studied compared to no ACE categories (ACE score of 0). One example of this pattern was perpetration of any IPV, where the odds ratio of perpetration compared to an individual with an ACE score of 0 increased steadily in magnitude from an ACE score of 1 (AOR 2.51; 95% CI: 1.46, 4.32) to an ACE score of 4 or more (AOR 11.52; 95% CI: 6.81, 19.51).

**Table 6:** Logistic regression analysis of the odds of intimate partner violence (IPV) perpetration with exposure to adverse childhood experiences (ACE) score among ever-partnered Sri Lankan men (n = 1252), UN Multi-Country Study on Men and Violence in Asia and the Pacific, Sri Lanka, 2011–2012.

ACE score	Emotional IPV		Financial IPV		Physical IPV		Sexual IPV		Any IPV	
	AOR^a^	(95% CI)	AOR^a^	(95% CI)	AOR^a^	(95% CI)	AOR^a^	(95% CI)	AOR^a^	(95% CI)
**0**	1.00	referent	1.00	referent	1.00	referent	1.00	referent	1.00	referent
**1**	2.67*	(1.36–5.25)	1.31	(0.53–3.26)	1.28	(0.58–2.81)	1.38	(0.53–3.60)	2.51*	(1.46–4.32)
**2**	4.70*	(2.40–9.22)	2.66*	(1.11–6.40)	2.56*	(1.20–5.45)	2.80*	(1.11–7.06)	4.07*	(2.35–7.04)
**3**	8.11*	(4.10–16.04)	4.03*	(1.69–9.59)	4.72*	(2.24–9.95)	4.00*	(1.59–10.08)	8.32*	(4.69–14.76)
**4 or more**	12.53*	(6.58–23.86)	6.35*	(2.83–14.23)	7.08*	(3.52–14.24)	5.72*	(2.38–13.72)	11.52*	(6.81–19.51)

^a^Adjusted odds ratio (AOR): adjusted for district, age group, and education level.

^b^95% confidence interval

*Identifies adjusted odds ratios that are statistically significant (p≤.05)

#### Relationships between Individual ACE Categories and IPV Perpetration

To identify their individual contributions to the increased odds of IPV perpetration, we included all eight ACE categories as separate variables in a logistic regression model for each IPV perpetration outcome, while controlling for district, age group, and educational attainment (Table 7). All three ACE categories of childhood abuse (emotional, physical, and sexual) as well as childhood hunger and childhood neglect were significant contributors to multiple forms of IPV perpetration. Childhood sexual abuse and childhood emotional abuse were significantly associated with perpetration of all five forms of IPV, with childhood sexual abuse associated with double the odds of sexual IPV perpetration (AOR 1.94; 95% CI: 1.31, 2.87) and any form of IPV perpetration (AOR 2.36; 95% CI: 1.69, 3.30). Childhood physical abuse was significantly associated with perpetration of three forms of IPV and was the highest childhood abuse contributor to the odds of physical IPV perpetration (AOR 1.74; 95% CI: 1.17, 2.59). Witnessing abuse of one’s mother was the strongest predictor of physical IPV perpetration (AOR 1.82; 95% CI: 1.29, 2.58). Peer victimization and lack of a male parental figure were the only two ACE categories that did not significantly contribute to any IPV perpetration regression model.

**Table 7:** Logistic regression analysis of the odds of intimate partner violence (IPV) perpetration with exposure to individual adverse childhood experiences (ACE) categories among ever-partnered Sri Lankan men (n = 1252), UN Multi-Country Study on Men and Violence in Asia and the Pacific, Sri Lanka, 2011–2012.

	Emotional IPV		Financial IPV		Physical IPV		Sexual IPV		Any IPV	
ACE category	AOR	(95% CI)	AOR	(95% CI)	AOR	(95% CI)	AOR	(95% CI)	AOR	(95% CI)
**Childhood emotional abuse**	1.80*	(1.30–2.48)	1.88*	(1.28–2.77)	1.72*	(1.20–2.47)	1.55*	(1.03–2.34)	1.69*	(1.20–2.36)
**Childhood physical abuse**	1.56*	(1.13–2.16)	1.02	(0.67–1.57)	1.74*	(1.17–2.59)	1.40	(0.88–2.23)	1.68*	(1.23–2.29)
**Childhood sexual abuse**	1.89*	(1.38–2.58)	1.88*	(1.27–2.78)	1.47*	(1.03–2.10)	1.94*	(1.31–2.87)	2.36*	(1.69–3.30)
**Childhood hunger**	1.45*	(1.09–1.93)	1.57*	(1.09–2.24)	1.71*	(1.24–2.36)	1.42	(0.98–2.05)	1.78*	(1.34–2.38)
**Childhood neglect**	1.50*	(1.12–2.02)	1.85*	(1.26–2.71)	0.92	(0.65–1.30)	1.47	(0.99–2.18)	1.49*	(1.11–2.01)
**Witnessed abuse of mother**	1.15	(0.83–1.58)	0.99	(0.67–1.45)	1.82*	(1.29–2.58)	1.05	(0.70–1.56)	1.17	(0.84–1.63)
**Lack of a male parental figure**	1.18	(0.86–1.60)	0.86	(0.59–1.26)	0.76	(0.53–1.09)	1.02	(0.67–1.53)	0.98	(0.72–1.34)
**Peer victimization**	1.04	(0.72–1.50)	1.08	(0.71–1.63)	1.45	(0.98–2.14)	1.20	(0.76–1.89)	0.96	(0.65–1.41)

^a^adjusted odds ratio (AOR): All logistic regression models and odds ratios adjusted for district, age group, and education level.

^b^95% confidence interval

*Identifies odds ratios that are statistically significant (α = .05)

## Discussion

Our study aimed to explore the relationship between Sri Lankan men’s ACEs and perpetration of emotional, financial, physical, and sexual IPV in adulthood. We found support for both our hypotheses. Sri Lankan men’s ACE scores (the cumulative number of ACE categories) had a positive dose-response relationship with all five of our IPV perpetration outcomes, similar to the relationship found in the US ACE study [27]. Additionally, examining individual ACE categories, we found that most ACE categories were significant contributors to one or more forms of IPV perpetration, which was consistent with other studies linking childhood abuse and witnessing abuse of one’s mother to perpetration of IPV in adulthood [20,23,24,33,35].

In our descriptive analyses, we identified high proportions of all forms of ACEs and IPV perpetration among men in Sri Lanka, with physical child abuse and emotional IPV perpetration most commonly reported. ACEs co-occurred in high numbers among the men, leading to high ACE scores. The high prevalence of physical child abuse in this sample mirrored the US ACE Study, where physical child abuse was the second most frequently reported ACE category (after substance abuse by a household member, which was not assessed in Sri Lanka). However, the prevalence of child physical abuse among Sri Lankan men (59%) was nearly three times the prevalence reported by American adults (22%) [28]. This difference in prevalence suggests that ACEs are more widespread in Sri Lanka, or at least much more likely to be reported by adults. We also found that all 8 ACEs were associated with every form of IPV perpetration before adjusting for co-occurrence, with education being the one covariate not associated with any form of IPV perpetration. The finding that education was not associated with IPV perpetration contrasts with previous findings showing education to be protective against perpetration of IPV [20,51]. This could simply be a result of low power in our sample, or it could highlight a difference in the effect of education in Sri Lanka on IPV perpetration compared to other countries.

Our multivariate regression results revealed that increases in cumulative ACEs were associated with increased odds of IPV perpetration, mirroring the US ACE study [27,28]. They also showed that sexual and emotional abuse had a more widespread association with the men’s likelihood to perpetrate IPV than the other forms of childhood abuse, while physical child abuse and witnessing abuse of one’s mother were the greatest contributors to the odds of physical IPV. These findings are supported by research conducted in the US and other countries showing links between childhood abuses, witnessing abuse of one’s mother, and IPV perpetration [27,28,37]. In contrast, peer victimization and lack of a male parental figure were not significant contributors to perpetration of any of the forms of IPV, suggesting that they did not have the same relationship with perpetration of IPV as the other ACE categories in Sri Lanka.

### Theoretical Implications

Researchers have developed theoretical frameworks to understand the factors that lead to IPV perpetration and identify ways to prevent it [52]. Bandura’s social learning theory of aggression is particularly relevant to our study, as he posited that individuals learn to behave violently when they perceive such behavior to be socially rewarded, meaning that boys could learn to perpetrate IPV by witnessing or experiencing a family member controlling others through violence [36]. The US ACE study’s findings provide supporting evidence for a social learning theory of IPV perpetration, as many of the ACE categories linked to perpetration of IPV involve abusive and violent behavior by adults [27,28]. In our study, the strong associations between experiencing childhood physical abuse and perpetrating physical IPV as an adult and between childhood sexual abuse and sexual IPV perpetration support a social learning theory of IPV perpetration; that is, men seemed to reproduce the violence they experienced as children against their partners in adulthood [36]. The association between witnessing abuse of one’s mother and perpetration of physical IPV also supports a social learning theory of IPV perpetration, since men in this category had witnessed adult men perpetrating physical IPV against their mothers in their childhood, and subsequently perpetrated physical IPV against their own female partners in adulthood. The relationship between social learning theory and IPV perpetration has been suggested by many IPV researchers in the past and is supported by our findings [37,53,54].

Peer victimization did not contribute significantly to any perpetration of IPV regression model. It was not associated with changes in perpetration of IPV in the same way as other ACE categories, and despite its value in American research [29], it might not be relevant to an ACE framework for understanding perpetration of IPV in Sri Lanka. Lack of a male parental figure also was not significantly associated with changes in the odds of perpetration of any form of IPV. When we examined interrelationships between ACE categories, lack of a male parental figure was significantly distributed only with a greater proportion of childhood emotional abuse. This lack of associations with other ACEs suggests that lack of a male parental figure in Sri Lanka might not be comparable to the separated or divorced parents category in the US ACE study and might be different from other ACEs in Sri Lanka in its distribution among and impact on young people [27]. However, in a 2008 review of US papers studying childhood experiences as risk factors for IPV perpetration, the authors found only one paper which included absent fathers as a risk factor and found no association between having an absent or rejecting father and IPV perpetration in adulthood [53,54]. This lack of evidence suggests that lack of a male parental figure as a risk factor for IPV perpetration needs to be further explored in a Western context as well.

### Strengths of this Study

Our study had unique strengths. The survey data came from over 1000 men from multiple regions of Sri Lanka, rather than only one region or city. Our study was the first to calculate a cumulative ACE score for Sri Lankan participants analogous to the score given to US ACE study participants and to incorporate peer victimization as an ACE category [28,29]. It was, therefore, the first study of the relationship between ACE scores and perpetration of four different forms of IPV among Sri Lankan men, as well as of the contributions of individual ACE categories to that relationship. This analysis of cumulative and overlapping ACEs adds to the general literature on ACEs and IPV perpetration, which often neglects to treat ACEs as co-occurring [37]. Our findings differentiated absence of a father figure in the Sri Lankan context from parental separation or divorce in the US framework, suggesting that female-headed households in Sri Lanka may be uniquely resilient. Finally, because various forms of ACEs occurred before perpetration of IPV as an adult, our findings highlighted a potential causal relationship between childhood abuse and adult violence that, if better understood, could be used to help develop interventions to protect both children and adults from family dysfunction. Previous studies of the effects of childhood abuses and witnessing IPV against one’s mother often focused on and found negative psychosocial outcomes in children but did not assess the additional effects in adulthood, as shown in Kitzman et al.’s review [26].

### Limitations and Future Research

This study also had some important limitations. First, the sample of men was not representative of all men in Sri Lanka, but only of men in the districts of Colombo, Hambantota, Batticaloa, and Nuwara Eliya. Future researchers should sample the entire country, not just certain districts, potentially by including questions about ACEs and IPV perpetration and victimization in the next national Demographic and Health Survey (DHS) questionnaire [50]. The survey methodology could also have introduced non-response bias, as men who were not available at home or refused to participate when the interviewers were conducting the household surveys might have been different from those who were available and chose to reply. The data were cross-sectional in nature and relied on participants accurately recalling and reporting their adverse childhood experiences and lifetime perpetration of IPV, both of which might have been impacted by social desirability bias during the interview. This is a common limitation in studies of IPV and it might have been minimized by the use of PDAs to allow participants to input sensitive answers alone [54]. The high reported prevalence of both ACEs and IPV perpetration suggest that men were comfortable with the approach used. Finally, the survey focused primarily on risk factors for perpetration of IPV and did not ask the participants about factors that could potentially promote resilience, such as supportive role models and relationships. This absence of data on resilience mirrors a lack of such data in the field of IPV research, and leading IPV researchers are now calling for a shift to focus on resilience, to help identify ways to prevent IPV [52]. It is critical that future researchers examine the role of positive factors, such as role models, strong relationships, and the development of gender equitable attitudes in a South Asian context (such as through the Program H intervention in India), particularly given the high rates of perpetration and acceptance of IPV in South Asian countries [15,55].

Finally, those interested in reducing IPV in Sri Lanka should examine the dynamics of single motherhood in Sri Lanka and how it differs from other ACE categories. Female-headed households increased dramatically during the 30-year Civil War [38]. However, prior studies have highlighted the heterogeneity of Sri Lanka’s female-headed households [56], which vary in ethnicity [57], relationship to war [58], and the stigma faced by the women [59]. The effects of these characteristics on future IPV perpetration of boys raised within female-headed households still needs to be assessed.

## Conclusion: Policy and Programmatic Implications

Our findings revealed a need to address the high rates of ACEs among Sri Lankan boys, as well as the association between ACEs and perpetration of IPV in adulthood. Policy and programmatic interventions to prevent ACEs could benefit children in the short term and have the added benefit of decreasing IPV when those children grow up and enter relationships. Because the most commonly reported physical abuse ACE in our sample was being beaten at school, the implementation of policies discouraging corporal punishment in Sri Lankan schools could help to greatly decrease ACEs in Sri Lanka. This form of violence has been termed “institutional battering” by Finkelhor and Korbin, and policies to decrease its prevalence could greatly improve child welfare [60].

Parenting programs have been shown to decrease child abuse and prevent IPV in high-income countries and may be a worthwhile intervention approach in low- and middle-income countries like Sri Lanka [61]. Many IPV prevention experts advocate developing resilience among children who have been exposed to ACEs to reduce the likelihood of these ACEs leading to violence [62–64]. Building resilience among young people could also have positive community-wide effects in Sri Lanka, which is still recovering from decades of civil war and the 2004 tsunami [38]. Investing in the well being of Sri Lanka’s young people could decrease the intergenerational transmission of trauma and violence and make the country a safer and less violent place for future generations.
